# Challenges and strategies in communication with people with dementia and their informal caregivers in community pharmacies – a narrative approach

**DOI:** 10.1111/scs.12789

**Published:** 2019-11-21

**Authors:** Katharina Heimerl, Barbara Pichler, Petra Plunger

**Affiliations:** ^1^ Institute for Palliative Care and Organizational Ethics Universtitaet Klagenfurt Wien Austria; ^2^ Department for Nursing Science University of Vienna Vienna Austria

**Keywords:** Dementia, care, informal caregivers, narrative approach, community pharmacy, stigmatisation, communication

## Abstract

**Background:**

People with dementia and their informal caregivers get in touch with the healthcare system predominantly via contacts with primary care providers. Among these, community pharmacists have been denominated as the health professionals most accessible to the public. Communication with and counselling people with dementia and their informal caregivers present particular challenges to pharmacists.

**Aim:**

This study aims to research the challenges faced and strategies used by community pharmacists who deal with people living with dementia and their informal caregivers.

**Methods:**

Within the context of two workshops with 74 participants, 15 small groups were formed, each of which generated and discussed a small story. Fourteen of those narratives were reported, tape recorded and transcribed. In these 14 narratives, community pharmacists reflected on their experiences with people with dementia or their informal caregivers. The narratives were systematically analysed and interpreted.

**Findings:**

Among the main challenges reported by the workshop participants are the difficulty of identifying a person with dementia; the question of what appropriate communication is; the only partially successful networking with doctors, nursing personnel and support institutions; unsuccessful counselling; and the tension between the economic situation and the care for people with dementia and their informal caregivers. In general, strategies for dealing with people with dementia are characterised by uncertainty whereas communication with informal caregivers is well rehearsed and effective.

**Conclusions:**

Community pharmacies require possibilities to retreat for counselling as well as the possibility for pharmacists to take time for people with dementia and their informal caregivers in everyday pharmacy life. Reflective spaces for narrations about difficult situations provide relief for staff in community pharmacies.

## Background

In Austria, there are about 130 000 people living with dementia, with an estimated 29 000 new diagnoses every year 1. Approximately 80% are cared for at home supported by family members, with 73% of informal caregivers being women 2.

People with dementia and their informal caregivers get in touch with the health care system predominantly via contacts with primary care providers 3. Among these, community pharmacists have been denominated as the health professionals most accessible to the general public 4. Community pharmacies have been described as easily accessible health care settings, responsible for the supply of medication and management of pharmacotherapy, but also for counselling 5, 6, 7, 8. Frequently, pharmacies provide the first contact within the healthcare system. They reach a broad spectrum of the population in their respective communities, including marginalised groups, and a disproportionately high share of older people and of women. Especially in rural areas, the percentage of regular customers is high. Pharmacists enjoy high levels of trust based on their expert status and competence.

As with other healthcare providers, dementia care has become more important in recent years in community pharmacies, for example, as partners in dementia‐friendly community initiatives 9.

However, there are some barriers regarding dementia care in the community pharmacy. Dementia is a stigmatised in society, which might cause resistance in pharmacy teams to deal with this issue 10. Furthermore, pharmacists have to deal with lack of privacy and time pressure 11. In addition, the perception of pharmacists as ‘shop keepers’ by clients and physicians might compromise dementia care and counselling activities 12. Only little do we know about everyday encounters of community pharmacists with people with dementia or with their informal caregivers.

## Methods

### Design

In this study, we aimed at gaining a deeper understanding of typical situations that involve people with dementia and their informal caregivers in community pharmacies. We wanted to explore the challenges that pharmacists experience in these situations and the strategies that guide the practical actions of the pharmacists. We therefore chose a qualitative research design; specifically a narrative approach for data collection 13 and the documentary method for data analysis 14.

To conduct the study, community pharmacists from three Austrian regions (Vienna, Lower Austria and the City of Salzburg) participated in a workshop. The workshop was held twice, the first one in Vienna and the second one in Salzburg. The workshops served a twofold purpose: (1) to collect data for the present study and (2) to start an intervention project aiming at implementing ‘dementia friendly pharmacies’. The latter project is descried elsewhere 15. We gained access to the field through the Austrian chamber of pharmacists, who acted as a project partner and picked the three regions. The chamber of pharmacists sent an invitation letter to all pharmacies in the three provinces to participate in the workshops and in the intervention project. Forty pharmacies accepted the invitation and participated in the workshops. These pharmacies agreed to delegate between 1 and 3 pharmacists to the project. Finally, all together 74 pharmacists participated in the two workshops of the present study. We expected the pharmacists to raise questions about better and more adequate ways to communicate with people with dementia and their informal caregivers. We therefore invited the coordinator and an informal caregiver of Alzheimer Austria – a self‐help group – to share their experiences with the pharmacists in the second part of each workshop.

### Data collection

In the two workshops, we collected oral narratives of the pharmacists’ professional experience. Like Riessmann 16 we see narratives as ‘storied ways of knowing and communicating’. Narratives have also been defined as ‘tools to understand, negotiate, and make sense of situations we encounter’ 17. Our concept of narrative refers to short and thematically specific stories that follow a plot with distinct characters in a distinct setting 13 to answer a specific research question. Contemporary discourses in narrative research in health care differentiate between big and small stories. The narratives we collected belong to the category of small stories 18, 19.

Table 1 shows the characteristics of the participants and the workshops.

**Table 1:** Characteristics of participating community pharmacy staff

Characteristics	Quantity
Number of participating pharmacies and pharmacists	40 pharmacies/74 participants
Participants per workshop	workshop 1: 40; workshop 2: 34
Group discussions per workshop	Workshop 1: 8; workshop 2: 7
Professions of participants	Pharmacists: 59; pharmaceutical sales assistant: 15
Gender	Men: 10; Women: 64
Position	Management: 21; Staff: 53
Participants per Austrian province	Vienna: 20; Lower Austria:20; Salzburg: 34

After an introduction to purpose and methods of the workshop, we randomly constituted small groups of four to five workshop participants. The task was to exchange individual stories and to choose one story that one group member was going to present in the plenary. To initiate the process of data collection, we wrote the following stimulus for the group discussion on a flip chart: ‘Please tell a story about a person with dementia or an informal caregiver in your pharmacy, which still preoccupied you later’. The chosen narrative should contain the following elements: (1) the story (what happened?), (2) reflection (what was it that preoccupied you?) and (3) solutions (What did you do?). We had used this three steps procedure successfully in an earlier project about ethical challenges in nursing homes 20.

### Data analysis

The documentary method 14 is based on Karl Mannheim’s sociology of knowledge, but also takes contributions from social phenomenology, ethnomethodology and symbolic interactionism. This methodology provides an understanding of collective orientations of subjects in a given social context, whose experiences are connected to a certain structure. The tacit or implicit knowledge that is the frame of orientation and which guides practical actions is represented in narrations and descriptions. Constitutive for the documentary method is the differentiation between communicative or explicit meaning and the conjunctive, implicit or documentary meaning. Practically, this differentiation leads to two successive steps of interpretation: formulating interpretation and reflecting interpretation.

Formulating Interpretation: The researcher formulates the explicit meaning of what the participants have said. The topical structure is reconstructed.

Reflecting Interpretation: This is a transition from asking ‘what’ to asking ‘how’. ‘What’ has been said is to be separated from ‘how’ (in which framework) the topic is dealt with. This framework of orientation is the central subject of documentary interpretation. By these means the documentary methods link thematic analysis to structural analysis, both described by Riessmann 13 as essential for analysing narrative interviews. It starts with the explication of the implicit frame of orientation (of the pharmacists) in the first case. In a ‘Case‐Internal Comparative Analysis’, we look for a homologous pattern in the same case or narrative. The next step is the ‘Comparative Analysis between Cases’. It is the first level of typification, or to use a term of Mannheim’s, ‘the meaning‐genetic’ 14 typification, in which the common frame of orientation (by comparing different cases) gets (re)constructed.

## Findings

Altogether, we collected 14 small stories that focus either on a person with dementia or on an informal caregiver or both. Each narrative focuses on a specific theme, as displayed in Table 2.

**Table 2:** Overview of narratives and themes

Number of Narrative	Focus	Theme
1	pwd – person with dementia	How to handle the drug dispenser box
2	pwd	The pharmacy as a florist
3	pwd	Problems paying and organising a taxi
4	pwd	How to administer drugs
5	pwd	How a well‐known patient changed her personality
6	Caregiver	Neighbour as caregiver and working network
7	Caregiver	The clock test
8	pwd and caregiver	Person with dementia destroyed plants
9	Caregiver	To involve the daughter in the care network
10	pwd	Customer does not know his daughter’s name
11	Caregiver and pwd	The pharmacy as a central place of help in a rural region
12	pwd	Calling the wife of a regular customer
13	pwd	Customer needs help with paying the bill
14	Caregiver	Advice in organising and accepting formal help

The stories have an explicit order of action that frames the encounters: almost all narratives start with a person with dementia or an informal caregiver entering the community pharmacy. These persons are perceived as acting subjects in the narratives, the pharmacy constitutes the surroundings and the pharmacists react. The storytellers then describe a challenging situation, very often followed by the pharmacist’s helplessness. Some narratives vary by including additional actors. The final part of the narrative focusses on accounts of strategies that the pharmacists use to find a solution for the challenging situation.

Further, in many cases the whole team is engaged in the search for a strategy. This is apparent in those parts of the stories where the storyteller indiscernibly slips from a first‐person narration to a ‘we’ narration.

That’s when I noticed there is also a certain communication problem. (…). And then there was a bit of a problem with the payment because she had enough money for the prescription fee, but not enough for the taxi that was apparently supposed to take her home, so we sent her to a, well, to the bank closest to us to withdraw money, but she was back after two minutes and said ‘Where was the bank, again’? And then we gave her the directions again. (N3, 205‐216).

There is no explicit mention of other colleagues being consulted for advice. Rather, in sensitive situations, actions of the individual pharmacist are embedded in collective action with the team, which is illustrated through the ‘we’‐form of narration. When pharmacists are at a loss for ways to deal with people with dementia, they turn to the team for help. However, there is no common understanding to use team reflection in these sensitive situations. Despite this collegial support in concrete situations, they do not explain how the group then discusses the situation. Instead, thinking about what has happened again becomes an individual matter.

### Challenges and strategies in communication with people with dementia

We asked about experiences with people with dementia and/or their informal caregivers in everyday pharmacy life, so dementia is the central topic in the stories. Nevertheless, participants rarely explicitly mention the word dementia. Only one of the 14 narratives mentions the term ‘onset of dementia’ (N2, 112). During the discussion, a pharmacy employee gives a reason for this cautious and precise use of language. Diagnosis is not within her tasks and competences and is an unknown practice in pharmacies.

I didn’t immediately diagnose dementia in my head, I’m not entitled to do that. (N1, 76‐77).

#### Recognising a person with dementia

What we found remarkable was that it wasn’t obvious when you looked at the customer. He was relatively young, about 60, and he actually seemed quite well. (N10, 109‐112).

This quote illustrates that it is usually not obvious at first glance, whether the person entering the pharmacy is a person with dementia. The interaction at the counter starts as usual, until the pharmacy employee first notices something unexpected and becomes more attentive. Sometimes, this can take time. For example, if someone fails to remember the name of the prescribed vitamins, but instead explains that they come in a brown bottle, this does not raise the suspicion of dementia. Behaviour only becomes noticeable for the employee if it is obvious that the person cannot cope with the current situation in the pharmacy or if a regular customer changes his/her usual behaviour. In the stories the pharmacy staff tell, the following situations serve as an indication that the customer is a person with dementia (Table 3).

**Table 3:** Criteria for identifying a person with dementia

Chaos in storage of drugs
Chaos in the wallet
Payment problems
Failure to understand explanations/advice
Counselling is unsuccessful
Word confusion
Failure to remember name of close relatives
Inappropriate behaviour: uprooting plants, aggressiveness
Prescription for Alzheimer’s drugs
Information from a third party, for example, the family doctor’s receptionist

#### Respectful Communication

Pharmacy staff express a need for more knowledge on how to communicate better and more adequately with this group of people. They also describe uncertainty in dealing with people with dementia and their informal caregivers. The stories document a strong effort of pharmacy staff to treat people with dignity and not to embarrass them. Such an unfortunate situation only occurs in one story, when the pharmacist does not realise that she is talking to a person with dementia, which she regrets immensely afterwards. If specific signs in a person's behaviour point towards dementia, they attempt to react appropriately. In most narratives, the person with dementia is overwhelmed with the current situation, which one pharmacist aptly describes as ‘double helplessness’:

So the first thing that catches your attention is the double helplessness. The patient is helpless because he just doesn't know what to do and we’re helpless for a short time because we just don't know how to react. (N13, 173‐176).

Taken in a positive light, this brief helplessness, this moment of not knowing how to proceed can also be considered a brief pause for thought. Therefore, while the course of action continues to be guided by the procedures described above, a more relaxed atmosphere can be created by adapting the conditions to the situation.

And then I had a problem finding the right choice of words, talk to her without being overbearing, without appearing condescending. The lady was very articulate, so for me it was not obvious at all. Then I went through the medication with her again and the advantage was that we have two armchairs on the side, so I could really sit down with her, that we could really go through the medication. (N4, 270‐277).

This quote illustrates the efforts being made to treat a person suspected of having dementia appropriately and respectfully. The pharmacist tries to adapt both her choice of words and the setting to the person's needs and the situation. At the same time, the resulting investment of time is also seen as a burden, as we will see below.

#### Unsuccessful counselling

Although in some situations, solutions are found – for example, by discreet assistance in the payment process – many questions about the success of counselling arise in pharmacists’ core business: even if advice on taking medication is well adapted to the situation, this usually does not lead to the desired outcome. The pharmacist is uncertain that the customer has really understood how to use his/her medication correctly at home.

Even though the exchange at the counter is relatively short, it does allow a degree of caring to develop. Once the pharmacy employee realises that a customer might be a person with dementia, she/he knows that the customer probably has trouble remembering things and, therefore, takes on the role of caregiver. Pharmacy staff then try to help within the scope of their possibilities. Nevertheless, staff in pharmacies is often left without knowing whether their counselling was successful.

So the big problem for me is the helplessness I feel in trying to ensure that she only takes what she’s supposed to take. After she’d left I also wondered whom I was allowed or duty bound to inform, or whom I should inform. I’m still not sure that she’s taking it correctly. (N4, 281‐285).

In the case of regular customers whose social networks are familiar to pharmacy staff, they take advantage of this knowledge to get in touch with customers’ informal caregivers and/or doctors. In the case that this cooperation is successful, it represents sustainable and appropriate problem‐solving.

#### Investment of time – between the economic, medical and psychosocial systems

Workshop participants describe several situations where they take time to patiently explain, write down or demonstrate correct drug administration, so that it is more comprehensible. The availability of a separate, quiet area for consultations makes this easier. An employee setting aside some time for a customer is seen as a luxury and not as standard practice. Sometimes, the customer comes when pharmacy staff are very busy, making it impossible for them to dedicate time for consultation.

The narratives clearly illustrate that dealing with people with dementia involves an increase in time allocation. When discussing this with participants, it becomes clear that pharmacy staff are constantly trying to fulfil different and contradictory requirements. This is most apparent in the expressed dilemma of dignified interaction versus efficient time management. Participants’ choice of words shows how strongly the economic factor features in this branch, in that they mostly refer to (regular) customers, thus clearly defining a business relationship. Consequently, pharmacy staff typically associate time with investment as can be seen in the following quote.

I’m patient, definitely invested a lot of time, at least half an hour, certainly 20 minutes, but it didn’t work. (N1, 44‐46).

Depending on how they perceive people who enter the pharmacy, staff tend to refer to them differently: for instance, seen from an economic perspective, they call them customers, whereas they call them patients, when they see them in a medical perspective. Sometimes, they refer to people as lady, woman, man, neighbour or relative, especially, if they are aware of the person’s social environment.

Time allocation is especially important when dealing with people with dementia, because as described, very often consultations are experienced as having been unsuccessful. Subsequently, pharmacists feel they have failed on two levels: on an economic level – the customer did not make a purchase and the pharmacist invested a lot of time, and on a professional level – the pharmacist was unable to uphold the quality they expect of themselves.

#### Uncertainty in strategies

The following quote shows a pharmacy employee explaining to a customer the correct way to place medical dosage for a week into a dispenser. Strategies for doing this – explanation, demonstration and labelling – all require time investment but still prove unsuccessful. Since she cannot solve the problem within the scope of her everyday tasks, she refers the customer back to the doctor who wrote the prescription, hereby delegating the problem elsewhere. The employee is left with the uncomfortable feeling that, once she is at home, the customer will not know how to manage her medication.

(…), so I wrote down on a piece of paper that she should take a whole tablet of drug X and half of drug Y. Then I realized we weren’t getting anywhere at all. I really invested a lot of time in explaining it to her, said to her ‚look, take one tablet of X and half of Y, on Tuesday take one tablet of X and half of Y’, then I said to her ‘and how would you carry on?’ and she managed to put tablet X in the right space for Wednesday morning, but she was already going to put a whole tablet Y in the box and I said to her ‘no, look, just half’. So, she doesn’t understand what belongs where. So, I started off by labelling the first compartment mornings, but in the end that didn’t work either. (N1, 26‐39).

Interaction with people that are assumed of having dementia often creates exceedingly challenging situations for pharmacy staff, since their usual course of action proves ineffective. While problems are identified very quickly and swiftly solved and tested, they do not always lead to the desired solutions. The procedure is linear: problem – solution – problem – solution – problem …

Although pharmacy staff actions are based on procedures set on a rational level, that is explanation, advice and demonstration, they do have alternatives at their disposal, for example, taking their time and finding a quiet place in the pharmacy for consultation. They describe these alternatives as appropriate interventions and problem‐solving.

There are also some successful interactions with people with dementia. In one case, an employee managed to help a man who had lost his wife, while she went for a walk in the city. However, even after successfully solved problems staff is aware that the things could have gone wrong. Pharmacy employees remain uncertain how to act and what to do the next time a similar situation arises.

### Challenges and strategies in persuading informal caregivers to seek support

Our comparisons between the narratives about informal caregivers, and the ones about people with dementia, reveal one fundamental difference. While dealing with people with dementia often leaves employees in pharmacies uncertain how to act and usual strategies prove ineffective, these well‐rehearsed strategies are usually successful with informal caregivers. This is expressed in the following story. In this narrative, the customer was a neighbour of the story‐telling pharmacist. The neighbour cared for one of the regular customers of the pharmacy.

Uncertainty is a problem, being able to give this neighbor a feeling of certainty, feel she can always come to us, ask questions. I explained again how to use everything and she said she’d been well informed by the doctor. She just wanted to hear it again from me. And when she left she was actually quite happy. (N6, 388‐393).

In this case, the neighbour who acted as an informal caregiver was unsure how to administer the prescribed medication and the pharmacist was able to react appropriately.

Apart from drug administration, consultation with pharmacy staff includes medical and psychosocial aspects, for example, they might advise a customer to see a neurologist or to seek professional support.

I think it is important to coax relatives to call a support service (…). To encourage people, go ahead and call. Look up a telephone number for them and tell them: here you are, just try. (N14, 310‐311; 331‐332)

These are challenging tasks for pharmacy staff, because they need to proceed with sensitivity and patience, as it takes time for informal caregivers to accept advice, they did not ask for and suggestions which are unrelated to drug administration. An existing customer relationship and pharmacy staff knowing doctors and support services are a prerequisite for this sort of advice. Although the latter is not generally the case, it proves to be an important source, when that knowledge is available.

### Challenges and strategies in networking with social and health professionals

Whether a contact with a person with dementia or their informal caregivers is successful and satisfying depends largely on the quality of local networks. Family doctors play a central role in these networks, but only when the relevant person is a regular customer and pharmacy employees know who their doctor is. On the one hand, doctors are consulted regarding prescriptions and diagnoses; on the other hand, they are the pharmacists’ obvious contact person regarding the organisation of nursing and care. In some cases, this cooperation works quite well.

I called the doctor. And he was, actually, very cooperative and said, ‚OK, interesting, I hadn’t noticed yet, but I will take a look’, because he anyway makes home visits to these two women and, of course he really did. He was very grateful that we had told him about it. (N2, 120‐123).

In this case, the pharmacy employee managed to tend to all aspects involved in an exchange with the customer. In addition to successful communication during the direct interaction, she was also able to reach the doctor, who agreed to keep an eye on the two women.

However, often, when contacting a doctor to explain that a person cannot manage the administration of their drugs pharmacy staff do not feel confident that this contact will lead to a solution:

Recently, I told the doctor that it wasn’t working. Well, the lady went home with her drug packets for intake the next day, as far as I remember, but I don’t think she can do it and I also don’t think she can take out the drugs. (N1, 47‐49).

Therefore, by delegating the problem to the doctor, the pharmacist passes on the responsibility. This, however, is not a sustainable method of problem‐solving and, thus, leaves the pharmacist unsatisfied with the situation.

Pharmacists often also know the social environment of their regular customers and keep records, which include telephone numbers. Thus, if problems arise, pharmacists can contact the informal caregivers. One employee of a pharmacy in the town centre gives the following example: a woman calls the pharmacist and asks him to contact her if he sees her husband is underway all alone. She also requests that the pharmacy staff help her husband find his way home if he happens to pass by the pharmacy.

Although family doctors are understood to play the central role in networks, there are also attempts to put informal caregivers in contact with support services. However, pharmacists are not the go‐between in these cases; instead, informal caregivers are advised to seek help from the support services directly.

When a person enters the pharmacy, who is not a regular customer, the pharmacists do not come to know, if their strategy worked. However, pharmacist staff continue to think about the situation and feel the need and the wish to know, for example, if the customer she called a taxi for made it home safely. They also wonder if they should have perhaps put an information brochure about Alzheimer’s disease in the shopping bag. In cases where unfamiliar customers display possible signs of dementia, there is no network to fall back on to ensure the person’s safety once they have left the pharmacy. Participants in the workshops of the present study have described this to be a burden.

## Critical interpretation and discussion

The stigmatisation of dementia and of people with dementia is an undisputed topic 21. The language used in public discussion is degrading and enhances stigmatisation 22. Our research is not the only one that shows how difficult it is for health professionals, to overcome the taboo and approach the topic of dementia with affected people appropriately. Healthcare professionals often find it challenging to talk about dementia to those living with dementia and their relatives 23. Frequently, communication with people with dementia takes place in settings of high time pressure such as hospitals, hereby creating particularly challenging situations for staff 24.

Community pharmacies are increasingly becoming places that offer health counselling. However, counselling people with dementia and their informal caregivers is particularly challenging for pharmacy staff 7. Likewise, in our study, the storytellers describe challenging situations, very often followed by helplessness of the pharmacist, although other moral emotions 25 are described. The main challenges are the difficulty of recognising a person with dementia; the question what appropriate communication is; the lack of formalised networks with family doctors and support services; unsuccessful consultation; and the difficulty of balancing economic aspects and good care. Generally, strategies for dealing with people with dementia are embedded in uncertainty.

Whereas situations with people with dementia are characterised by uncertainty, there is much more certainty in strategies for dealing with informal caregivers, despite the challenges involved in convincing them to accept help and even though counselling informal caregivers is time‐consuming. Prior research has shown that other health professionals also feel informal caregivers use up too much of their time and take up their resources. This leads health professionals to perceive informal caregivers as an obstacle in their daily work 26.

Communication with people with dementia and their informal caregivers is challenging for community pharmacy staff. Staff seek strategies primarily on the individual level. Although the analysis reveals that social and organisational issues are important, they are not in the sight of the pharmacists. On a structural level, pharmacies would need service providers who are experienced with dementia with whom they can form networks.

The pharmacists participated in the group discussion voluntarily. All participants of the group discussions subsequently participated in a participatory research project 27 with the title ‘Dementia friendly pharmacy’ 28. They were highly motivated and expressive about psychological stress and the wish to work on it in the following project. Some of the participants were themselves caregivers of people with dementia. We consider it a limitation of our study that this particular group of narrators constituted a positive selection. While we do not presume that all pharmacy staff are convinced of the importance of dealing with people with dementia and their informal caregivers to the same extent, the stories show that it is increasingly becoming an important factor in community pharmacies.

In order to utilise the findings from the group discussions for the processes and activities of the project ‘Dementia friendly pharmacies’, we also asked about solutions, rather than concentrating exclusively on problem definition. In the following project, pharmacists could work on those questions that were left unanswered in the narratives. Several dilemmas faced by the pharmacists – especially that of reconciling limited time resources in the work place with the time‐consuming communication with people with dementia and their informal caregivers – remain. The very definition of pharmacy as an enterprise aiming to maximise profit on the one hand and a healthcare provider with the duty to provide good care for vulnerable people on the other, makes this reconciliation particularly important, albeit difficult.

Ferlie and Shortell 29 identify the following four levels of change for improving quality in health care: individual, team, organisation and the wider system.

While it is possible to achieve a small, limited impact by focusing only on one of the four levels for change, we believe that the greatest and long‐lasting impact will be achieved by considering all four levels simultaneously ( 29 , 288).

In our study, the pharmacists primarily addressed the individual level: they see it as their responsibility to develop individual strategies to deal with the challenges associated with communication with people with dementia and their informal caregivers. Educational trainings conveying a person‐centred approach to communication 30 to staff in community pharmacies widen their strategies to deal with challenging situations with people with dementia on an individual level.

Only when they threaten to fail, community pharmacy staff switch to the ‘narrating we’ in their narratives, which means that they seek solutions on the team level in the respective pharmacy. As our study shows, community pharmacy staff welcome the possibility to share and reflect their experiences with people with dementia and their informal caregivers.

Pharmacists experience a tension between the individual and the team level on one hand and the organisational level on the other hand: time constraints due to the organisational characteristics of community pharmacies are perceived as hindering factors for communication with people with dementia and their informal caregivers.

Our research focused mainly on the individual, team and organisational levels. However, there were also clues pointing at the system level, for example, when pharmacy staff narrated their contact with physicians and their efforts to convince caregivers to make use of support services. Our study is limited as it did not reveal many aspects regarding the wider system such as the question how the health care system needs to be organised in order to alleviate time constraints and support community pharmacists to adopt and implement a person‐centred attitude towards people with dementia and their informal caregivers.

## Conclusion

Community pharmacies have the potential to become ‘dementia friendly’. This would require changes on the individual, the team, the organisational and the wider system level. Community pharmacy staff need education in person‐centred care in order to deal better with challenging situations with people with dementia and their informal caregivers. Furthermore, better networking with doctors, support services and self‐help groups who are specialised in supporting people with dementia and their informal caregivers would enable community pharmacy staff to refer their customers to adequate services. Teams in community pharmacies need reflective spaces where they can share their experiences, difficulties and strategies in dealing with challenging situations with people with dementia and their informal caregivers. These skills and knowledge on the individual pharmacists’ and the team level will only be effective if community pharmacies implement organisational changes such as creating places of retreat for consultation as well as time slots for people with dementia and their informal caregivers. Further research is required that investigates how changes on the wider system level could support community pharmacies in becoming ‘dementia friendly’.

## Conflict of interest

There is no conflict of interest to disclose.

## Authors contribution

P.P. and K.H. managed the project with the support of B.P.; K.H. developed the workshop design and facilitated the group discussions with the support of P.P. and B.P; P.P. and B.P. managed the data; B.P. analysed the data with the help of K.H. and P.P.; B.P. drafted the chapter ‘findings’; P.P. drafted the chapter ‘background’; and K.H. wrote the manuscript. All authors discussed the results and contributed to the draft and the final manuscript.

## Ethical considerations

As the project’s aim was focused on professional development and on organisation development, we did not seek approval by the ethical committee. Furthermore, participating staff of community pharmacies were not considered to be extraordinary vulnerable. However, the research was approved by the chamber of pharmacists and adhered to the quality standards of the Austrian Health Promotion Foundation.

Pharmacists volunteered to participate in the project. We informed the participating pharmacists about the project, its aims and methodology and their role both in a written project information prior to the project and orally in the beginning of the workshop. The pharmacists gave their explicit verbal consent about notes and audio recording of the workshops and about the use of the data for research. Confidentiality was guaranteed by anonymising the transcripts. It was left to the individual pharmacist to decide, whether they wanted to disclose their narrative to the plenary. As some of the participants also had experiences as informal caregivers, we offered broad space for discussion and enabled for reflection of the individual experiences, if a participant wished to do so. In the plenary discussion, a member of Alzheimer Austria, the Austrian self‐help group for caregivers of people with dementia, was present to answer any questions, participants might have about communication with people with dementia.

## Funding

The research was funded by the Austrian Health Promotion Foundation (Fonds Gesundes Österreich); the Vienna Health Promotion Foundation (Wiener Gesundheitsförderung); and the Austrian Chamber of Pharmacists (Österreichische Apothekerkammer).

## References

[1] Wancata J . Studien und Hochrechnungen für Österreich (Studies and projections for Austria) In Österreichischer Demenzbericht 2014 (Austrian Dementia Report 2014) (Höfler S, Bengough T, Winkler P, Griebler R eds), 2015, Bundesministerium für Gesundheit und Sozialministerium, Wien 19–21.

[2] Nagl‐Cupal M , Kolland F , Zartler U , Mayer H , Bittner M , Koller M , Parisot V , Stöhr D . Angehörigenpflege in Österreich: Einsicht in die Situation pflegender Angehöriger und in die Entwicklung informeller Pflegenetzwerke (Informal Care by relatives in Austria. Insights in the situation of caregivers and the development of informal care networks), 2018, Bundesministerium für Arbeit, Soziales, Gesundheit und Konsumentenschutz, Wien.

[3] Spenceley SM , Sedgwick N , Keenan J . Dementia care in the context of primary care reform: an integrative review. Aging Ment Health 2015; 19: 107–20. 24901364 10.1080/13607863.2014.920301

[4] World Health Organisation (WHO) . The role of the pharmacist in the health care system. 1994 http://apps.who.int/medicinedocs/en/d/Jh2995e/ (last accesses 7 January 2019).

[5] Anderson C , Blenkinsopp A , Armstrong M . Feedback from community pharmacy users on the contribution of community pharmacy to improving the public's health: a systematic review of the peer reviewed and non‐peer reviewed literature 1990–2002. Health Expect 2004; 7: 191–202. 15327458 10.1111/j.1369-7625.2004.00274.x PMC5060240

[6] del Mar Alonso‐Perales M , Lasheras B , Beitia G , Beltrán I , Marcos B , Núñez‐Córdoba JM . Barriers to promote cardiovascular health in community pharmacies: a systematic review. Health Promot Int 2017; 32: 535–48. 26511943 10.1093/heapro/dav098

[7] Eades CE , Ferguson JS , O'Carroll RE . Public health in community pharmacy: a systematic review of pharmacist and consumer views. BMC Public Health 2011; 11: 582. 21777456 10.1186/1471-2458-11-582 PMC3146877

[8] Plunger P . The Role of the pharmacist in health promotion: Experiences from a European project. Framacevtski Vestnik 2000; 51: 181–6.

[9] Alzheimer´s Society . Resources for Pharmacists. https://www.alzheimers.org.uk/dementia-professionals/resources-professionals/resources-pharmacists (last accessed 21 January 2019).

[10] Rubio‐Valera M , Chen TF , O'Reilly CL . New roles for pharmacists in community mental health care: a narrative review. Int J Environ Res Public Health 2014; 11: 10967–90. 25337943 10.3390/ijerph111010967 PMC4211017

[11] Joyce AW , Sunderland VB , Burrows S , McManus A , Howat P , Maycock B . Community pharmacy's role in promoting healthy behaviours. J Pharm Pract Res 2007; 37: 42–44.

[12] Barry HE , Parsons C , Passmore AP , Hughes CM . Community pharmacists and people with dementia: a cross‐sectional survey exploring experiences, attitudes, and knowledge of pain and its management. Int J Geriatr Psychiatry 2013; 28: 1077–85. 23348866 10.1002/gps.3931

[13] Riessman CK . Narrative methods for the human sciences. 2008, Sage, Los Angeles.

[14] Bohnsack R . Documentary Method In The SAGE Handbook of Qualitative Data Analysis. (Flick U ed), 2014, Sage, London, 217–33.

[15] Plunger P , Heimerl K , Tatzer VC , Zepke G , Finsterwald M , Pichler B Reitinger E . Developing dementia‐friendly pharmacies in Austria: A health promotion approach. Health Promot Int 2019: 1–12. 10.1093/heapro/daz063 31292603

[16] Riessman CK . Narrative Analysis In Narrative, memory and everyday life, (Kelly N, Horrocks C, Milnes K, Roberts B, Robinson D, ed.), 2005, University of Huddersfield, Huddersfield 1–7.

[17] Adams TE . A review of narrative ethics. Qual Inq 2008; 14: 175–94.

[18] Bamberg M , Georgakopoulou A . Small stories as a new perspective in narrative and identity analysis. Text Talk 2008; 28: 377–96.

[19] Sools A . Narrative health research: exploring big and small stories as analytical tools. Health 2013; 17: 93–110. 22694917 10.1177/1363459312447259

[20] Reitinger E , Heimerl K . Ethics and gender issues in palliative care in nursing homes: an Austrian participatory research project. Int J Older People Nurs 2014; 9: 131–9. 24862993 10.1111/opn.12049

[21] van Gorp B , Vercruysse T . Frames and counter‐frames giving meaning to dementia: a framing analysis of media content. Soc Sci Med 2012; 74: 1274–81. 22386639 10.1016/j.socscimed.2011.12.045

[22] Johnstone M‐J . Metaphors, stigma and the 'Alzheimerization' of the euthanasia debate. Dementia 2013; 12: 377–93. 24336950 10.1177/1471301211429168

[23] Hansen A , Hauge S , Bergland Å . Balancing the use of language to enable care: a qualitative study of oral and written language used in assessments and allocations of community healthcare services for persons with dementia. BMC Health Serv Res 2016; 16: 391. 27530603 10.1186/s12913-016-1659-0 PMC4988005

[24] Allwood R , Pilnick A , O'Brien R , Goldberg S , Harwood RH , Beeke S . Should I stay or should I go? How healthcare professionals close encounters with people with dementia in the acute hospital setting. Soc Sci Med 2017; 191: 212–25. 28934622 10.1016/j.socscimed.2017.09.014 PMC5630221

[25] Haidt J . The moral emotions In Handbook of Affective Sciences (Davidson RJ, Scherer KR, Goldsmith HH eds), 2003, Oxford University Press, Oxford 852–70.

[26] Laursen J , Broholm M , Rosenberg J . Health professionals perceive teamwork with relatives as an obstacle in their daily work ‐ a focus group interview. Scand J Caring Sci 2017; 31: 547–53. 27862154 10.1111/scs.12368

[27] Hockley J , Froggatt K , Heimerl K (eds). Participatory research in palliative care: actions and reflections. 2013, Oxford University Press, Oxford.

[28] Plunger P , Tatzer V , Heimerl K , Reitinger E . Dementia‐friendly pharmacy: A doorway in the community in Vienna and Lower Austria In Compassionate communities: Case studies from Britain and Europe (Wegleitner K, Heimerl K, Kellehear A, eds), 2015, Routledge, London, New York. 137–52.

[29] Ferlie EB , Shortell SM . Improving the quality of health care in the United Kingdom and the United States: a framework for change. Milbank Q 2001; 79: 281–315. 11439467 10.1111/1468-0009.00206 PMC2751188

[30] Kitwood TM . Dementia reconsidered: The person comes first. 1997 Open University Press, Maidenhead, England.

